# Challenges and Opportunities of Early Parkinson’s Disease Biomarkers: α-Synuclein, Leucine-Rich Repeat Kinase 2 (LRRK2), DJ-1, and microRNAs

**DOI:** 10.7759/cureus.91510

**Published:** 2025-09-02

**Authors:** Allie Heineman, Jillian Linck, Adi Eylon, Mayur S Parmar

**Affiliations:** 1 Osteopathic Medicine, Nova Southeastern University Dr. Kiran C. Patel College of Osteopathic Medicine, Clearwater, USA; 2 Osteopathic Medicine, Nova Southeastern University Dr. Kiran C. Patel College of Osteopathic Medicine, Fort Lauderdale, USA; 3 Neurology, Memorial Healthcare System, Pembroke Pines, USA; 4 Foundational Sciences, Nova Southeastern University Dr. Kiran C. Patel College of Osteopathic Medicine, Clearwater, USA

**Keywords:** alpha-synuclein, dj-1, lrrk2, micrornas (mirnas), parkinson-plus syndromes, parkinson’s disease (pd), serum biomarker

## Abstract

Parkinson’s disease (PD) is a progressive neurodegenerative disorder characterized by dopaminergic neuron loss, leading to motor symptoms (tremor, rigidity, bradykinesia, postural instability) and non-motor symptoms (autonomic dysfunction, cognitive dysfunction, olfactory dysfunction, mood disorders such as depression). Pathologically, PD involves Lewy bodies, aggregates of phosphorylated α-synuclein (α-Syn) driven by genetic or environmental factors. α-Syn, detectable in cerebrospinal fluid (CSF), blood, and saliva, is a promising biomarker for early diagnosis and monitoring. Other candidates, including leucine-rich repeat kinase 2 (LRRK2), DJ-1, and microRNAs (miRNAs), reflect genetic, oxidative stress, and gene regulatory changes in PD. Ideal biomarkers should be sensitive, specific, non-invasive, and track disease progression. However, PD’s heterogeneity, biofluid variability (e.g., hemolysis), methodological inconsistencies, and the need for large-scale validation pose challenges. Standardization of sample collection, assays, and cohort characterization is critical for clinical application. This review evaluates α-Syn, LRRK2, DJ-1, and miRNAs, highlighting their strengths, limitations, and potential. Advanced assays targeting specific α-Syn species, LRRK2 kinase activity, oxidized DJ-1, and miRNA panels, alongside non-invasive matrices (saliva, skin, retina) and multi-biomarker approaches, offer significant opportunities for early detection and therapy monitoring, warranting further research and standardization.

## Introduction and background

Parkinson’s disease (PD) is a neurodegenerative disorder characterized by progressive loss of dopaminergic neurons in the nucleus of the substantia nigra pars compacta of the basal ganglia, a critical area of the brain involved in movement control. This dopaminergic neuronal loss results in the classic motor symptoms of PD, such as resting tremor, rigidity, akinesia, bradykinesia, and postural instability [1,2]. Pathologically, PD is characterized by the presence of Lewy bodies, resulting from the aggregation of phosphorylated alpha-synuclein (α-syn) proteins caused by genetic mutations or other environmental factors [3,4]. These α-syn aggregates are believed to be harmful because they disrupt normal cellular functions, leading to neuronal dysfunction and death.

While the loss of dopaminergic neurons is a hallmark of PD, it is important to note that some degree of neuronal loss can also occur in healthy aging, though to a lesser extent. It has been associated with the development of Parkinsonian symptoms in advanced age. The normal age-related changes in neuroprotective cellular signaling include reduced neurotrophic factors, increased oxidative stress, dysfunction in intracellular Rab GTPase-associated trafficking (a process vital for transporting substances within cells), mitochondrial dysfunction, and other factors [2,4-7]. These changes are associated with the PD neuropathology. According to the Parkinson's Foundation, approximately 1.1 million people are living with PD in the United States, with nearly 90,000 new cases diagnosed each year, and over 10 million people impacted worldwide [8]. This significant societal burden underscores the urgency of improved diagnostic and therapeutic strategies.

Current PD medications primarily provide symptomatic relief, managing motor symptoms, such as tremors, rigidity, and bradykinesia, but do not halt or reverse disease progression [9,10]. Due to the significant challenges in treating PD, multiple drug developments are underway to identify disease-modifying therapies that can mitigate the progression of PD by targeting pathophysiology, such as the α-syn aggregation in the brain [11,12]. The major challenge in developing disease-modifying therapies is identifying the patient early so the intervention can be approached on time [13]. The importance of early identification cannot be overstated; many disease-modifying therapies tested in clinical trials for neurodegenerative diseases, including PD, fail because, by the time drug intervention occurs, the disease has often progressed too far for reversal. Furthermore, non-motor symptoms such as sleep disturbances, loss of smell, and constipation often appear years before motor symptoms and are crucial for early diagnosis. Therefore, identifying specific and sensitive biomarkers for PD can help lead to an earlier diagnosis and initiation of disease-modifying therapies. These biomarkers can also allow researchers to evaluate the therapeutic effects of reducing pathophysiology during the clinical trial phases [12,14].

A promising biomarker for PD should ideally meet several key criteria. First, it should be sensitive and specific, meaning it can accurately distinguish between PD patients and healthy controls and differentiate PD from other neurological disorders [15]. Second, the biomarker should be non-invasive and easy to measure [15]. It should be available through a simple, safe procedure such as blood drawing. Finally, the biomarker should correlate with disease progression [15]. This means that it should be able to track the progression of PD over time and predict future outcomes. These criteria are crucial in identifying an effective biomarker for PD. However, developing a biomarker for PD presents several challenges. PD is a complex neurodegenerative disease with multiple contributing factors, such as genetic mutations, environmental toxins, and aging [9,11], making it challenging to identify a single, sensitive, and specific biomarker. The disease also exhibits heterogeneity, with patients presenting a wide range of symptoms and progression rates, complicating the development of a universally applicable biomarker [16]. Furthermore, despite extensive research, the underlying mechanisms of PD remain poorly understood, hindering the identification of biomarkers that accurately reflect the disease process. Finally, developing and validating biomarkers necessitates large-scale studies involving well-characterized cohorts of patients with PD and healthy controls. These studies are often costly and time-consuming, highlighting the intricate and resource-intensive biomarker development process [16].

This comprehensive narrative review aims to highlight specific and sensitive PD biomarkers under preclinical and clinical evaluation. Potential biomarkers include measuring the levels of α-syn (in cerebrospinal fluid (CSF), blood, saliva, and tissue samples), microRNAs (miRNAs), and genetic markers such as DJ-1 and leucine-rich repeat kinase 2 (LRRK2). These potential biomarkers were reviewed due to substantial evidence of disease association and preliminary data supporting their detection in biochemical assays.

## Review

Literature search strategies

A comprehensive literature search was conducted using databases including PubMed, Google Scholar, and Web of Science. The search focused on publications from the past 25 years (2000-2025), using combinations of key terms such as “Parkinson’s disease”, “PD biomarkers”, “alpha-synuclein”, “LRRK2”, “DJ-1”, “miRNAs”, and “polygenic risk score”. To ensure historical context and completeness, three foundational papers published prior to 2000 were also included, as they represent seminal discoveries in the field. Particular emphasis was placed on literature published between 2020 and mid-2025 to capture the most recent advancements in PD biomarker research and genetic profiling.

Overview of potential biomarkers

α-Synuclein

α-Synuclein (α-syn), a 140-amino-acid presynaptic neuronal protein, is critical for synaptic function but plays a central role in PD pathogenesis due to its propensity to aggregate and form Lewy bodies, the hallmark of PD and related α-synucleinopathies, such as multiple system atrophy (MSA) and dementia with Lewy bodies (DLB) [11,17]. Structurally, α-syn is typically unfolded or adopts an α-helical conformation when membrane-bound under physiological conditions, but, in PD, it misfolds into β-sheet-rich oligomers - the most neurotoxic form-disrupting presynaptic vesicle trafficking and dopamine neurotransmission [18,19]. In advanced disease, these oligomers progress to insoluble fibrils with cross-β-sheet architecture, seeding further aggregation and forming Lewy bodies [19]. Genetic mutations in the SNCA gene, which encodes α-syn, cause familial PD, underscoring its pathogenic significance [20]. As a key protein in PD pathology, α-syn is a promising biomarker, detectable in peripheral fluids such as blood and CSF, enabling early diagnosis and monitoring of disease progression [21-23].

CSF α-Syn Biomarkers

CSF biomarkers have been extensively studied in α-syn research, revealing strengths and limitations over decades of investigation. Measuring α-syn levels in bodily fluids and tissues has emerged as a promising strategy for diagnosing PD and differentiating it from other α-synucleinopathies. Several techniques have been developed to quantify α-syn in CSF, including enzyme-linked immunosorbent assay (ELISA), electrochemiluminescence, and multiplex assays [24]. Several studies have shown that patients with PD have significantly lower levels of total α-syn in their CSF than healthy individuals [24,25]. However, these findings can vary, especially in cases of PD dementia (PDD) and DLB, where total α-syn levels may not differ significantly from controls. Nonetheless, elevated levels of α-syn oligomers have been observed in PDD and DLB patients compared to controls and Alzheimer's disease (AD) cases, suggesting greater relevance of oligomeric or pathological species [26].

The observed reduction in CSF α-syn levels is hypothesized to result from increased intracellular aggregation, neuronal loss, or decreased SNCA gene expression in response to impaired exocytosis of α-syn [27]. Decreased α-syn levels in the CSF can be a useful biomarker tool for PD, as indicated by research findings [28]. The study involved analysis of CSF samples from 299 individuals (comprising patients with PD, AD, and healthy controls); α-syn concentrations in these samples were quantified using Luminex assays, while methods such as Western blot contributed to initial protein characterization. The study found significantly reduced CSF α-syn levels in PD patients, with 92% sensitivity and 58% specificity in individuals aged ≥65 years when controlling for age and blood contamination. Despite high sensitivity, it is important to interpret this modest specificity with caution.

More recently, advances in CSF-based α-syn detection, such as the seed amplification assay (SAA), have shown significant promise. In the most extensive study of this kind to date, involving 1,123 subjects across eight countries, a cross-sectional study demonstrated the feasibility of using CSF α-syn SAA to differentiate PD from controls [16]. This study reported an overall sensitivity of 88% for participants with PD and a specificity of 96.3% for healthy controls. Interestingly, sensitivity in this study was lower in participants carrying pathogenic LRRK2 mutations, suggesting a potentially different etiological pathway in this subgroup, whereas sensitivity rose to 99% in patients with an olfactory deficit. Additionally, a positive CSF α-syn SAA was detected in 86% of at-risk individuals with rapid eye movement (REM) sleep behavior disorder (RBD) or hyposmia, highlighting its strong utility for identifying the prodromal stages of PD [16]. Longitudinal follow-up will be important to understand this test's clinical application further.

*Blood α-Syn* *Biomarkers*

Blood biomarkers are preferred over CSF biomarkers due to their easy accessibility. The potential transport of α-syn from the central nervous system (CNS) to the blood suggests that blood fluid biomarkers may reflect α-syn levels [29]. Numerous studies have attempted to measure serum and plasma α-syn levels, but results remain inconsistent. Some report elevated α-syn in PD, others report reductions, and several show no significant difference from controls [30-32]. These discrepancies may be partly due to hemolysis, as erythrocytes are rich in α-syn and can contaminate plasma/serum samples. One study tested 53 patients who were not taking PD treatment and 42 patients who were taking L-DOPA and found an increase in α-syn levels in both groups using ELISA [31]. In contrast, another study reported no significant differences in serum α-syn levels between PD patients and controls [32].

While measurements of total α-syn in blood have yielded inconsistent results, recent advancements targeting specific α-syn species and amplification techniques have shown significant promise for PD diagnosis and prodromal detection. Serum L1CAM-positive extracellular vesicle (L1EV) α-syn, assessed through electrochemiluminescence, effectively differentiated individuals with isolated RBD (iRBD) from controls (AUC = 0.91) [33]. It also distinguished those with a greater than 80% likelihood of prodromal PD from those with less than 5% probability (AUC = 0.80). Notably, elevated levels were observed in 80% of at-risk individuals who subsequently developed PD or associated dementia [33]. Similarly, a blood-based α-syn SAA detected pathological α-syn conformers in peripheral blood up to 10 years prior to clinical PD diagnosis in all 12 participants later diagnosed with PD [23]. Moreover, 30% of individuals with iRBD tested positive, while all healthy controls tested negative, highlighting its potential as a prodromal diagnostic biomarker. Building on this, an immunoprecipitation-based real-time quaking-induced conversion (IP/RT-QuIC) method was used to concentrate and amplify α-syn from serum [34]. The study detected pathological α-syn in 95% of PD patients, 90% of DLB patients, and 44% of iRBD patients, with electron microscopy revealing distinct filament structures in PD and DLB sera compared to multiple system atrophy (MSA). This further supports its utility for non-invasive diagnosis and differential diagnosis of α-synucleinopathies. These findings underscore the potential of targeted blood-based α-syn assays to enhance early detection and risk stratification for PD and related disorders. This is especially important since early symptoms such as constipation, RBD, and anxiety can appear over 20 years before a formal diagnosis [35].

In peripheral blood, erythrocytes represent another promising matrix. A study using electrochemiluminescence quantified total, aggregated, and phosphorylated (pS129) α-syn in erythrocyte membranes and cytosolic fractions [36]. Total and aggregated α-syn levels were found to be significantly higher in PD erythrocyte membrane fragments than in controls. Furthermore, in PD subjects, pS129 levels were remarkably higher in the cytosolic fraction and, to a lesser extent, in the membrane fraction. By combining age, aggregated α-syn in erythrocytic membranes, and cytosolic pS129 levels, a model created through logistic regression analysis distinguished patients with PD from neurologically normal controls, achieving a sensitivity of 72% and specificity of 68% [36]. These results suggest erythrocytic α-syn, especially pS129, as a peripheral biomarker candidate requiring further validation.

Salivary α-Syn Biomarkers

Saliva has emerged as a promising non-invasive biofluid for PD biomarker development due to its ease of collection, cost-effectiveness, and the potential for CNS pathology to be reflected in the innervation and secretions of the salivary glands. The pathology of α-syn is known to affect peripheral autonomic nerves, including those innervating the salivary glands, which may result in detectable pathological α-syn species in saliva. Supporting its potential for early detection, studies have shown elevated α-syn in the saliva of PD patients and, importantly, in prodromal cases. A recent study demonstrated a significant increase in total α-syn within salivary small extracellular vesicles (sEVs) in PD patients (with 88.24% sensitivity) and prodromal cases compared to healthy controls, highlighting its promise as a biomarker for detection potentially up to 20 years prior to formal diagnosis [37].

Recent research has further investigated advanced methodologies, such as α-syn SAA in saliva. The presence of α-syn SAA in both saliva and serum was examined, showing that, while saliva α-syn SAA independently demonstrated promising outcomes for diagnosing PD, integrating serum α-syn SAA significantly enhanced diagnostic efficacy [38]. This achieved a high sensitivity of 95.83% and specificity of 96.15%, comparable to CSF α-syn SAA. Furthermore, the study indicated potential correlations between these seeding activities and various clinical measures [38]. Further exploring combined salivary biomarkers, a study demonstrated that pairing salivary α-Syn RT-QuIC SAA with salivary miRNA-29a-3p (decreased in PD and MSA) enhanced diagnostic sensitivity and specificity for PD compared to healthy controls. This combined approach also improved effectiveness in differentiating PD and MSA from essential tremor and offered increased specificity for distinguishing PD from MSA [39].

The findings emphasize saliva's increasing utility as a source of biomarkers; however, certain challenges persist, including low baseline α-syn concentrations, susceptibility to oral contamination, and variability in sample quality. Nonetheless, developing sensitive amplification assays and multiplexed strategies continues to validate saliva’s potential as a clinically significant and non-invasive matrix for diagnosing and differentiating PD.

*Skin α-Syn* *Biomarkers*

Beyond fluids such as CSF and blood, peripheral tissue sources, including skin, have emerged as viable sites for detecting pathological α-syn. Skin biopsies are minimally invasive, well-tolerated, and have already been used in clinical neurology for diagnosing small fiber neuropathies, making them attractive for biomarker development [40]. Importantly, α-syn aggregates have been observed in autonomic and somatosensory nerve fibers within the skin in α-synucleinopathies, offering a biological rationale for their diagnostic use [41,42].

A recent cross-sectional study investigated the detection of phosphorylated α-syn in skin biopsies from patients with a clinical diagnosis of PD, DLB, MSA, and pure autonomic failure (PAF) and control participants [42]. Using immunohistochemical detection, the study found a high detection rate of phosphorylated α-syn in skin biopsies of patients with α-synucleinopathies: PD (92.7%), MSA (98.2%), DLB (96.0%), and PAF (100%). In contrast, only 3.3% of the control group without α-synucleinopathies had detectable phosphorylated α-syn in their skin biopsies. These findings support the potential of phosphorylated α-syn in the skin as a clinically relevant diagnostic marker. However, additional research is necessary in unselected clinical populations to validate these findings.

Further findings demonstrated that skin biopsies accurately differentiated PD and non-PD patients through RT-QuIC and protein misfolding cyclic amplification (PMCA) assays (RT-QuIC showing 95% sensitivity and 100% specificity for PD in these antemortem samples) [43]. Biopsies for these analyses on living patients were taken from the posterior cervical and leg sites of individuals with and without PD [43]. These skin-based assays' high sensitivity and specificity, particularly RT-QuIC, suggest that they could significantly influence clinical practice by enabling accurate, non-invasive diagnosis of PD and related α-synucleinopathies, potentially facilitating earlier intervention and differential diagnosis. However, broader validation in diverse clinical populations and the standardization of biopsy and assay protocols are necessary.

Retinal α-Syn Biomarkers

The retina has attracted increasing interest as a potential site for non-invasive biomarker development in PD [44]. As an extension of the CNS, the retina shares embryological origin with the brain and contains dopaminergic neurons, making it a rational site to explore for neurodegenerative pathology such as α-synucleinopathies [45,46]. Interestingly, several clinical trials are underway to evaluate the retinal functional changes in PD [44].

Studies analyzing the retinas of autopsied subjects with PD, incidental Lewy body disease (ILBD), and healthy controls using immunohistochemistry have revealed the presence of phosphorylated α-syn in the retinas of all PD and most (three of four) ILBD patients. Furthermore, the amount of retinal α-syn correlated with the severity of brain pathology, suggesting that retinal α-syn could be an early indicator of disease progression [47]. These findings underscore the retina’s potential as a non-invasive biomarker source, although further validation in living individuals is needed to translate this approach into clinical practice. A comprehensive postmortem study further confirmed p-α-syn (pSer129) as Lewy neurites in the inner plexiform layer of PD retinas and oligodendroglial cytoplasmic inclusions in the optic nerve of MSA cases, with no retinal Lewy neurites in MSA. This achieved high specificity (97%) and sensitivity (82%) for distinguishing primary α-synucleinopathies from other neurodegenerative diseases and controls, suggesting potential for differential diagnosis [48]. Additionally, a recent study using electroretinography (ERG) in M83 transgenic mice and early-stage PD patients detected reduced scotopic b-wave, photopic b-wave, and photopic negative response (PhNR) amplitudes, particularly in females [49]. This reduction was accompanied by p-α-syn accumulation in the inner nuclear and outer plexiform layers of the retina, suggesting early functional impairments associated with α-syn pathology [49]. These findings underscore the retina’s potential as a window into central α-syn pathology. However, the current evidence base is largely derived from postmortem samples, and further research using in vivo imaging or biopsy-based techniques in living individuals is needed to validate its translational utility.

Despite these advances, current assays that measure total α-syn levels directly in CSF, blood, saliva, and other tissues have often lacked the specificity and sensitivity required for diagnosing PD and require further exploration. However, it is essential to differentiate these findings: traditional assays measuring total α-syn levels directly in CSF, blood, saliva, and other tissues have often lacked the specificity and sensitivity required for definitive PD diagnosis and require further exploration. Newer generation assays targeting specific forms of α-syn (e.g., L1EV α-syn or phosphorylated α-syn in the skin) or employing amplification techniques (e.g., skin RT-QuIC/PMCA) are demonstrating very high sensitivity and specificity.

In summary, α-syn is a central molecular player in PD pathogenesis and holds immense promise as a diagnostic and prognostic biomarker. Table 1 summarizes various findings from studies evaluating α-syn as a potential biomarker in PD. Although challenges persist in optimizing and standardizing assays, especially for total α-syn in bulk biofluids, current research focuses on different bodily fluids and tissues. This research particularly targets specific α-syn species and utilizes advanced detection techniques. As a result, it provides considerable promise for enhancing early detection and tracking the progression of PD.

**Table 1:** α-Synuclein biomarkers for Parkinson's disease CSF: cerebrospinal fluid; DLB: dementia with Lewy bodies; ELISA: enzyme-linked immunosorbent assay; MSA: multiple system atrophy; PAF: pure autonomic failure; PD: Parkinson’s disease; PMCA: protein misfolding cyclic amplification; RT-QuIC: real-time quaking-induced conversion

Study	Study Subjects	Method Evaluated	Biomarker	Key Findings
Cerebrospinal Fluid Biomarkers				
Mollenhauer et al., 2019 [24]; Eusebi et al., 2017 [25]	PD patients and healthy individuals.	Measurement of total α-syn in CSF (e.g., ELISA, electrochemiluminescence, multiplex assays).	Total α-syn in CSF.	Patients with PD have significantly lower levels of total α-syn in their CSF than healthy individuals.
Hansson et al., 2014 [26]	Patients with Parkinson’s Disease Dementia (PDD), Dementia with Lewy Bodies (DLB), controls, and Alzheimer's disease (AD) cases.	Measurement of α-syn oligomers in CSF.	α-Syn oligomers in CSF.	Elevated levels of α-syn oligomers observed in PDD and DLB patients compared to controls and AD cases. Total α-syn levels may not differ significantly from controls in PDD and DLB.
Hong et al., 2010 [28]	299 individuals (patients with PD, AD, and healthy controls).	Quantification of α-syn concentrations in CSF samples using Luminex assays (Western blot for initial protein characterization).	Total α-syn in CSF.	Significantly reduced CSF α-syn levels in PD patients. Achieved 92% sensitivity and 58% specificity in individuals aged ≥65 years (controlling for age and blood contamination).
Siderowf et al., 2023 [16]	1,123 subjects across eight countries (including participants with PD, healthy controls, at-risk individuals with RBD or hyposmia).	CSF α-syn SAA.	Pathological α-syn seeding activity in CSF.	Overall sensitivity of 88% for PD participants and specificity of 96.3% for healthy controls. Positive CSF α-syn SAA in 86% of at-risk individuals (RBD or hyposmia). Sensitivity lower in LRRK2 mutation carriers, higher (99%) in patients with olfactory deficit.
Blood Biomarkers				
Duran et al., 2010 [31]	53 PD patients not taking treatment, 42 PD patients taking L-DOPA.	ELISA assays to measure α-Syn levels in plasma/serum.	Total α-syn in plasma/serum.	Increase in α-syn levels found in both untreated and L-dopa-treated PD patient groups.
Yan et al., 2024 [33]	Individuals with isolated REM sleep behavior disorder (iRBD), controls, individuals with high/low probability of prodromal PD, and at-risk individuals.	Measurement of serum L1CAM-positive extracellular vesicle (L1EV) α-Syn via electrochemiluminescence.	Serum L1EV α-syn.	Distinguished iRBD from controls (AUC = 0.91). Distinguished high probability prodromal PD from low probability (AUC = 0.80). Elevated levels in 80% of at-risk individuals who later developed PD or related dementia.
Kluge et al., 2024 [23]	Participants later diagnosed with PD (n=12), iRBD individuals, healthy controls.	Blood-based α-syn-SAA.	Pathological α-syn conformers in peripheral blood.	Detected pathological α-syn conformers up to 10 years before clinical PD diagnosis in all 12 participants later diagnosed. 30% of iRBD individuals tested positive; all healthy controls negative.
Okuzumi et al., 2023 [34]	PD patients, Dementia with Lewy Bodies (DLB) patients, iRBD patients.	IP/RT-QuIC method to concentrate and amplify α-syn from serum; electron microscopy.	Pathological α-Syn in serum; distinct filament structures.	Detected pathological α-Syn in 95% of PD patients, 90% of DLB patients, and 44% of iRBD patients. Electron microscopy revealed distinct filament structures in PD and DLB sera compared to MSA.
Tian et al., 2019 [36]	PD patients and neurologically normal controls.	Electrochemiluminescence to quantify total, aggregated, and phosphorylated (pS129) α-Syn in erythrocyte membranes and cytosolic fractions.	Total, aggregated, and pS129 α-Syn in erythrocytes.	Total and aggregated α-syn levels significantly higher in PD erythrocyte membrane fragments. pS129 levels are remarkably higher in the PD cytosolic fraction. Model (age, aggregated α-syn in membranes, cytosolic pS129) distinguished PD from controls with 72% sensitivity and 68% specificity.
Salivary Biomarkers				
Wang et al., 2024 [38]	PD patients (implied from the context of diagnostic efficacy).	α-Syn-SAA in saliva and serum.	α-Syn-SAA seeding activity in saliva and serum.	Saliva α-syn-SAA showed promising outcomes for PD diagnosis. Integration with serum α-syn-SAA significantly enhanced diagnostic efficacy (sensitivity 95.83%, specificity 96.15%), comparable to CSF α-syn-SAA. Potential correlations with clinical measures.
Luan et al., 2024 [39]	PD patients, MSA patients, essential tremor patients, healthy controls.	Salivary α-syn RT-QuIC SAA combined with salivary miRNA-29a-3p measurement.	Salivary α-syn RT-QuIC SAA and salivary miRNA-29a-3p.	Combined approach enhanced diagnostic sensitivity and specificity for PD vs. healthy controls. Improved differentiation of PD/MSA from essential tremor. Increased specificity for distinguishing PD from MSA.
Skin Biomarkers				
Gibbons et al., 2024 [42]	Patients with PD, DLB, MSA, pure autonomic failure (PAF), and control participants.	Immunohistochemical detection in skin biopsies.	Phosphorylated α-syn in skin biopsies.	High detection rate of phosphorylated α-Syn in skin biopsies of patients with α-Synucleinopathies: PD (92.7%), MSA (98.2%), DLB (96.0%), PAF (100%). Only 3.3% of controls had detectable phosphorylated α-syn.
Wang et al., 2020 [43]	PD patients and non-PD patients/individuals without PD (controls).	RT-QuIC and PMCA assays on skin biopsies (antemortem samples).	Pathological α-Syn aggregation seeding activity in skin.	RT-QuIC showed 95% sensitivity and 100% specificity for PD in antemortem skin samples. Accurately differentiated PD and non-PD patients.
Retinal Biomarkers				
Ortuño-Lizarán et al., 2018 [47]	Autopsied subjects with PD, incidental Lewy body disease (ILBD), and healthy controls.	Immunohistochemistry on retinas of autopsied subjects.	Phosphorylated α-Syn in the retina.	Phosphorylated α-syn present in the retinas of all PD subjects and most (3 of 4) ILBD patients. Amount of retinal α-syn correlated with the severity of brain pathology.
Hart de Ruyter et al., 2023 [48]	Postmortem cases of PD, MSA, other neurodegenerative diseases, and controls.	Postmortem analysis of retina and optic nerve (immunohistochemistry implied).	p-α-Syn (pSer129) as Lewy neurites (retina) and oligodendroglial cytoplasmic inclusions (optic nerve).	p-α-Syn (pSer129) as Lewy neurites in the inner plexiform layer of PD retinas. Oligodendroglial cytoplasmic inclusions in the optic nerve of MSA cases. No retinal Lewy neurites in MSA. High specificity (97%) and sensitivity (82%) for distinguishing primary α-Synucleinopathies.
Soto Linan et al., 2025 [49]	M83 transgenic mice and early-stage PD patients.	Electroretinography (ERG); p-α-syn immunohistochemistry in retina (mice implied).	ERG wave amplitudes (scotopic b-wave, photopic b-wave, PhNR); p-α-Syn accumulation in retina.	Reduced scotopic b-wave, photopic b-wave, and PhNR amplitudes in M83 mice and early PD patients (esp. females). p-α-Syn accumulation in the retina’s inner nuclear and outer plexiform layers (mice implied).

Leucine-rich repeat kinase 2 (LRRK2)

LRRK2 Genetic Mutations and Risk

LRRK2, located at the Park8 locus on chromosome 12q12, harbors significant genetic mutations associated with familial and sporadic autosomal dominant PD [50]. Several point mutations, notably G2019S, affect the enzymatic domain, often increasing kinase activity and disrupting cellular signaling. LRRK2 mutations are more prevalent in specific populations, such as North African Berber and Ashkenazi Jews, with G2019S present in 1-2% of clinically sporadic cases globally and higher in these groups [51]. Elevated LRRK2 kinase activity is observed in subsets of idiopathic PD, suggesting a broader role beyond genetic mutations [52].

In a large study involving researchers from 21 centers worldwide and 24 populations, the risk of developing PD for a person who inherits a specific LRRK2 point mutation (Gly2019Ser) was found to be significant. Specifically, the risk was 28% at age 59, 51% at age 69, and 74% at age 79. These findings raise the possibility that identifying this genetic mutation could serve as a potential diagnostic biomarker for PD [53]. These findings highlight LRRK2’s role as a genetic risk indicator, particularly in high-risk populations.

CSF LRRK2 Levels

Quantifying LRRK2 in CSF has emerged as a potential pharmacodynamic biomarker for PD. A novel methodology, the stable isotope standard captured by antipeptide antibody (SISCAPA) assay, quantified LRRK2 levels with high sensitivity compared to traditional methods, such as extracellular vesicle enrichment and Western blot detection. Patients with PD carrying the G2019S LRRK2 mutation exhibited significantly higher CSF LRRK2 levels than healthy controls, sporadic PD patients, and G2019S LRRK2 non-manifesting carriers, supporting the hypothesis that mutated LRRK2’s pathogenicity stems from overabundance and overactivity [54]. This suggests CSF LRRK2 as a potential biomarker for LRRK2-targeting therapies, though it requires further validation for clinical reproducibility and relevance.

Peripheral Blood LRRK2 Biomarkers

Peripheral blood offers an accessible source for LRRK2 biomarker development, particularly for kinase activity. A high-throughput flow cytometry-based assay (WHOPPA) detected elevated LRRK2 kinase activity via increased pT73-Rab10 phosphorylation in monocytes from idiopathic and GBA-associated PD patients compared to controls, supporting peripheral blood as a viable biomarker source [55]. Research indicates that pT73-Rab10 is elevated in peripheral blood mononuclear cells (PBMCs) from idiopathic PD (iPD) patients and affected G2019S-LRRK2 carriers, but not in A53T-α-syn PD patients, detected by Western immunoblotting [56]. In urinary EVs from iPD patients, cross-sectional studies found unchanged pT73-Rab10 levels compared to controls [56,57], but a longitudinal analysis revealed increasing pT73-Rab10 over time, correlating with iPD disease severity, though not in G2019S-LRRK2 carriers [57]. However, LRRK2 autophosphorylation (pS1292-LRRK2) was often undetectable in iPD urinary EVs [56]. These studies did not provide new evidence regarding its elevation in iPD PBMCs. However, the combined findings strongly indicate that pT73-Rab10 in PBMCs and its dynamic rise in urinary EVs are promising pharmacodynamic markers for LRRK2 inhibitors, especially in iPD [56,57]. These assays highlight LRRK2’s broader role in PD beyond genetic mutations, with potential for clinical trial stratification and therapy monitoring.

LRRK2 Prodromal and Cellular Biomarkers

LRRK2’s predictive potential is evident in prodromal and cellular studies. Eleven percent of LRRK2 non-manifesting carriers had dopamine transporter (DaT) deficits, with carriers showing higher Movement Disorders Society Unified Parkinson’s Disease Rating Scale scores and hyposmia rates than controls, suggesting LRRK2 as a predictive biomarker for PD development [58]. Complementing these findings, LRRK2-mediated centrosomal cohesion deficits in peripheral lymphoblastoid cells from G2019S LRRK2-PD patients, reversible by LRRK2 inhibitors, propose a novel cellular biomarker for patient stratification in LRRK2-targeted therapies [59]. Preclinical insights further elucidate mechanisms, with LRRK2 mutant mice showing increased susceptibility to motor defects and dopaminergic neuron loss due to gut inflammation via the tumor necrosis factor alpha (TNF-α) pathway, linking environmental and genetic risk [60].

LRRK2 Neuropathological and Prognosis Biomarkers

LRRK2 mutations are associated with diverse neuropathological outcomes in PD. The Parkinson’s Progression Marker Initiative’s brain autopsy program reported severe Lewy body disease in all LRRK2 mutation carriers among 17 brain donations [61]. However, other studies indicate variable pathology, including typical Lewy bodies, tau pathology, or nigral degeneration without Lewy bodies, suggesting LRRK2 mutations may predict specific PD sequelae [59,62-64]. These findings position LRRK2 as a potential prognostic biomarker for tailoring therapeutic strategies.

In summary, LRRK2 mutations play a significant role in PD, with genetic testing, CSF levels, peripheral blood assays, prodromal signs, and neuropathological variations providing distinct insights. Table 2 summarizes various findings from studies evaluating LRRK2 as a potential biomarker in PD. Furthermore, novel assays detecting pT73-Rab10 and pS1292-LRRK2 phosphorylation in peripheral biofluids and centrosomal deficits in cells could enhance LRRK2’s utility for patient stratification and therapy monitoring. Additionally, these biomarkers may facilitate earlier disease detection and support clinical trial design, particularly for non-mutation carriers. However, clinically validated biomarkers for tracking LRRK2 function are still needed, underscoring the importance of ongoing research to clarify LRRK2’s role in PD pathogenesis and improve patient outcomes.

**Table 2:** LRRK2 biomarkers for Parkinson's disease CSF: cerebrospinal fluid; LRRK2: leucine-rich repeat kinase 2; PBMC: peripheral blood mononuclear cells; PPMI: Parkinson’s Progression Markers Initiative

Study	Study Subjects	Method Evaluated	Biomarker	Key Findings
Genetic Biomarkers				
Di Maio et al., 2018 [52]	Subsets of idiopathic PD patients.	Proximity ligation assays to assess Ser1292 phosphorylation of LRRK2 and, separately, the dissociation of 14-3-3 proteins from LRRK2.	LRRK2 kinase activity.	Wild-type LRRK2 kinase activity is selectively enhanced in substantia nigra dopamine neurons in iPD patients, suggesting a role for LRRK2 kinase inhibitors in treating iPD without LRRK2 mutations.
Healy et al., 2008 [53]	Individuals from 21 centers worldwide, 24 populations (carriers of LRRK2 Gly2019Ser mutation and controls implied).	Genetic analysis for LRRK2 Gly2019Ser mutation and penetrance assessment.	LRRK2 point mutation (Gly2019Ser).	Risk of developing PD for G2019S carriers was 28% at age 59, 51% at age 69, and 74% at age 79. Highlights LRRK2 as a genetic risk indicator.
Cerebrospinal Fluid Biomarkers				
Mabrouk et al., 2020 [54]	PD patients with G2019S LRRK2 mutation, healthy controls, sporadic PD patients, LRRK2 G2019S non-manifesting carriers.	Stable Isotope Standard Captured by Antipeptide Antibody (SISCAPA) assay for LRRK2 quantification in CSF.	LRRK2 levels in CSF.	Significantly higher CSF LRRK2 levels in PD patients with G2019S LRRK2 mutation compared to healthy controls, sporadic PD patients, and LRRK2 G2019S non-manifesting carriers.
Peripheral Blood Biomarkers				
Wallings et al., 2022 [55]	Monocytes from idiopathic and GBA-associated PD patients and controls.	High-throughput flow cytometry-based assay (WHOPPA).	LRRK2 kinase activity (via pT73-Rab10 phosphorylation in monocytes).	Detected elevated LRRK2 kinase activity via increased pT73-Rab10 phosphorylation in monocytes from idiopathic and GBA-associated PD patients compared to controls.
Petropoulou-Vathi et al., 2022 [56]	Idiopathic PD (iPD) patients, affected G2019S-LRRK2 carriers, A53T-α-Syn PD patients, (controls implied for PBMC; iPD for urinary EVs).	Western immunoblotting (for pT73-Rab10 in PBMCs); Assays for pS1292-LRRK2 and pT73-Rab10 in urinary EVs.	pT73-Rab10 in PBMCs; pS1292-LRRK2 in urinary EVs; pT73-Rab10 in urinary EVs (cross-sectional).	pT73-Rab10 elevated in PBMCs from iPD patients and affected G2019S-LRRK2 carriers (not in A53T-α-Syn PD). pS1292-LRRK2 often undetectable in iPD urinary EVs. Unchanged pT73-Rab10 in urinary EVs from iPD (cross-sectional).
Wang et al., 2022 [57]	iPD patients, G2019S-LRRK2 carriers, (controls implied for urinary EVs).	Assays for pT73-Rab10 in urinary EVs (cross-sectional and longitudinal analysis).	pT73-Rab10 in urinary EVs (cross-sectional and longitudinal).	Unchanged pT73-Rab10 levels in urinary EVs from iPD patients compared to controls (cross-sectional). Longitudinal analysis revealed increasing pT73-Rab10 over time in urinary EVs, correlating with iPD disease severity (not in G2019S-LRRK2 carriers).
Prodromal and Cellular Biomarkers				
Simuni et al., 2020 [58]	LRRK2 non-manifesting carriers, controls.	Dopamine transporter (DaT) imaging, Movement Disorders Society Unified Parkinson’s Disease Rating Scale (MDS-UPDRS), hyposmia assessment.	DaT deficits, MDS-UPDRS scores, hyposmia rates (in LRRK2 carriers).	11% of LRRK2 non-manifesting carriers had DaT deficits. Carriers showed higher MDS-UPDRS scores and hyposmia rates than controls. Suggests LRRK2 (mutation status) as a predictive biomarker.
Naaldijk et al., 2024 [59]	Peripheral lymphoblastoid cells from G2019S LRRK2-PD patients.	Cellular biology assays for centrosomal cohesion.	LRRK2-mediated centrosomal cohesion deficits.	Centrosomal cohesion deficits in peripheral lymphoblastoid cells from G2019S LRRK2-PD patients, reversible by LRRK2 inhibitors. Proposed as a cellular biomarker for patient stratification.
Neuropathological and Prognosis Biomarkers				
Bukhari et al., 2022 [61]	17 brain donations from LRRK2 mutation carriers (PPMI brain autopsy program).	Brain autopsy, neuropathological assessment.	Neuropathology (Lewy body disease).	Reported severe Lewy body disease in all LRRK2 mutation carriers among 17 brain donations.
Zimprich et al., 2004 [62]; Ross et al., 2006 [63]; Taymans et al., 2010 [64]	Referenced studies of LRRK2 mutation carriers.	Neuropathological assessments in various studies.	Neuropathological features (Lewy bodies, tau pathology, nigral degeneration).	These studies indicate variable neuropathology in LRRK2 mutation carriers, including typical Lewy bodies, tau pathology, or nigral degeneration without Lewy bodies. Suggests LRRK2 mutations may predict specific PD sequelae.

DJ-1

DJ-1, also known as PARK7, is a highly conserved redox-sensitive protein composed of 189 amino acids and encoded by the PARK7 gene on chromosome 1p36. DJ-1 can suppress the production of reactive oxygen species (ROS) through various mechanisms, inhibit cellular apoptosis, and regulate multiple transcription factors, which activate protective pathways against oxidative stress [65]. Oxidative stress is a critical mediator in PD, as evidenced by increased levels of oxidation products in nigral cells [66,67]. Ongoing research is exploring whether DJ-1 may serve as a biomarker for PD.

Oxidized DJ-1 Levels

Many studies have focused on an oxidized DJ-1 protein as a reflection of overall oxidative stress [66]. A significant twofold increase in oxidized DJ-1 levels was observed in the urine exosomes of Korean patients with PD compared to controls, as measured by ELISA [68]. Focusing on oxidized DJ-1 may provide more relevant information than measuring total DJ-1, as it directly reflects a critical pathological process. In another study, higher levels of DJ-1 were also found in the urine of Korean males with PD, as previously noted [69]. However, no significant differences were found in the urine of Korean females with PD [69]. While DJ-1, especially in its oxidized form, shows potential as a biomarker for PD, its specificity may be limited. This is because oxidative stress markers such as oxidized DJ-1 can also be elevated in other neurodegenerative or systemic oxidative conditions. Therefore, further studies with rigorous methodological controls are necessary to validate its clinical utility.

Cerebrospinal Fluid (CSF) DJ-1 Levels

Several other studies have explored DJ-1 levels in CSF. Increased DJ-1 levels were reported in the CSF of patients with early-stage PD, suggesting that DJ-1 may serve as a potential early diagnostic marker [70]. However, in a study using the Luminex bead-based assay with controls for blood contamination, decreased DJ-1 levels were found in the CSF of PD patients [28]. Their method demonstrated 90% sensitivity and 70% specificity for distinguishing PD from controls in individuals aged ≥65 years, and 92% sensitivity and 55% specificity for differentiating PD from AD in the same age group [28]. These contradictory findings are likely influenced by methodological differences, such as the presence or absence of blood contamination controls, which can significantly impact DJ-1 measurement due to its high abundance in red blood cells.

DJ-1 Isoforms

In addition to changes in total concentration, DJ-1 isoforms in whole blood have been investigated for their biomarker potential in PD. Two-dimensional gel electrophoresis coupled with mass spectrometry was employed to identify and quantify these isoforms [71]. The research indicated that specific post-translationally modified DJ-1 isoforms, particularly those with 4-hydroxy-2-nonenal (4-HNE) modifications, demonstrated significant variations related to the stage of PD. Notably, the fraction of HNE-modified DJ-1 isoform 4 tended to decrease as PD severity increased, a finding that reached statistical significance in late-stage PD patients compared to controls and early-stage PD. Conversely, the fraction of HNE-modified DJ-1 isoform 6 (relative to the total HNE-modified DJ-1 pool) increased in late-stage PD patients. These distinct changes in specific, oxidatively modified DJ-1 isoforms in whole blood support their potential as stage-specific biomarkers for PD.

Serum and Plasma DJ-1 Levels

Using serum and plasma to measure DJ-1 levels and comparing these levels with those of healthy controls has produced inconsistent results. In plasma samples from sporadic PD patients, higher DJ-1 levels were observed, as determined by Western blot and ELISA techniques [70]. Furthermore, in patients with advanced-stage PD, DJ-1 levels were significantly elevated compared to those in early-stage patients. However, no significant differences in serum DJ-1 protein levels were found in Chinese patients with PD compared to healthy controls [72].

This study aims to confirm the findings of Maita et al., who similarly reported no significant differences in DJ-1 serum levels between Japanese PD patients and healthy controls [73]. These inconsistencies are often attributed to variations in sample types (plasma versus serum), differences in measurement methods (e.g., ELISA versus immunoblotting), and potential contamination from red blood cells.

DJ-1 in Neuron-Derived Exosome

The concept of quantifying DJ-1 in neuron-derived exosomes isolated from plasma was introduced using an immunoprecipitation-based method to control for contamination from erythrocytes, which are known to have high DJ-1 levels [74]. The study was the first to examine DJ-1 in this context and found significantly increased levels of both DJ-1 and α-syn in PD patients compared to healthy controls in these exosomes. Notably, a significant positive correlation between the levels of DJ-1 and α-syn in the exosomes of PD patients was observed, suggesting a shared pathological mechanism. The study's findings highlight the potential of DJ-1 and α-syn in plasma neural-derived exosomes as non-invasive biomarkers for diagnosing and monitoring PD.

In summary, DJ-1 shows promise as a multifaceted PD biomarker across urine, CSF, blood, and exosomes, particularly in its oxidized and isoform-specific forms. Table 3 summarizes various findings from studies evaluating DJ-1 as a potential biomarker in PD. However, inconsistencies due to biological variability, methodological differences, and contamination issues highlight the need for standardized, specific assays. Future validation studies are critical to establishing DJ-1’s clinical utility in diagnosing and monitoring PD.

**Table 3:** DJ-1 biomarkers for Parkinson's disease ELISA: enzyme-linked immunosorbent assay; HNE: 4-Hydroxynonenal; PD: Parkinson's disease

Study	Study Subjects	Method Evaluated	Biomarker	Key Findings
Oxidized DJ-1				
Jang et al., 2018 [68]	Korean PD patients and controls.	ELISA to quantify oxidized DJ-1 levels in urine exosomes.	Oxidized DJ-1 in urine exosomes.	Significantly higher (twofold increase) oxidized DJ-1 in urine exosomes of Korean PD patients compared to controls.
Ho et al., 2014 [69]	Korean males and females with PD.	Western blot analysis measures total DJ-1in urine.	Total DJ-1 in urine.	Significantly higher levels of DJ-1 found in the urine of Korean males with PD. No significant differences in the urine of Korean females with PD.
CSF DJ-1 Levels				
Waragai et al., 2006 [70]	Patients with early-stage PD.	Western blot analysis to measure total DJ-1 in CSF	Total DJ-1 in CSF.	Reported increased DJ-1 levels in the CSF of patients with early-stage PD.
Hong et al., 2010 [28]	PD patients, AD patients, and controls (individuals aged ≥65 years highlighted).	Luminex bead-based assay for DJ-1 in CSF (with controls for blood contamination).	Total DJ-1 in CSF.	Found decreased DJ-1 levels in the CSF of PD patients. Method demonstrated 90% sensitivity and 70% specificity for PD vs. controls (≥65 yrs), and 92% sensitivity and 55% specificity for PD vs. AD (≥65 yrs).
DJ-1 Isoforms				
Lin et al., 2012 [71]	PD patients (early and late-stage), controls.	2-D gel electrophoresis coupled with mass spectrometry to identify and quantify DJ-1 isoforms in whole blood.	Post-translationally modified DJ-1 isoforms (esp. 4-HNE-modified) in whole blood.	Fraction of HNE-modified DJ-1 isoform 4 tended to decrease as PD severity increased (significant in late-stage PD). Fraction of HNE-modified DJ-1 isoform 6 increased in late-stage PD patients. Potential as stage-specific biomarkers.
Serum and Plasma DJ-1 Levels				
Waragai et al., 2006 [70]	Sporadic PD patients (early and advanced-stage).	Western Blot and ELISA to measure total DJ-1 in plasma.	Total DJ-1 in plasma.	Plasma samples from sporadic PD patients were associated with a higher DJ-1 level. In advanced-stage PD patients, DJ-1 levels were more elevated compared to early-stage patients.
An et al., 2018 [72]	Chinese patients with PD and controls.	Western blot and ELISA to measure DJ-1 protein levels in serum.	Serum DJ-1 protein levels.	No significant differences in serum DJ-1 protein levels between Chinese PD patients and controls.
Maita et al., 2008 [73]	Japanese PD patients and controls.	ELISA kit to measure DJ-1 in serum.	Serum DJ-1 levels.	No significant differences in DJ-1 serum levels between Japanese PD patients and controls.
DJ-1 in Neuron-Derived Exosomes				
Zhao et al., 2018 [74]	PD patients and healthy controls.	Immunoprecipitation-based method to quantify DJ-1 (and α-syn) in neuron-derived exosomes isolated from plasma.	DJ-1 (and α-syn) in plasma neural-derived exosomes.	Significantly increased levels of both DJ-1 and α-Syn in plasma neural-derived exosomes of PD patients compared to healthy controls. Significant positive correlation between DJ-1 and α-Syn levels in exosomes of PD patients.

microRNAs (miRNAs)

miRNAs, small noncoding RNAs involved in gene regulation, have gained attention as potential biomarkers for PD due to their altered expression in disease states. miRNAs are small noncoding RNAs of approximately 20 nucleotides that suppress mRNA expression through translation inhibition, degradation, or deadenylation [75]. Chronic inflammation of the CNS is mediated by microglial cells and plays a significant role in PD pathogenesis [76]. Therefore, post-transcriptional regulation of gene expression of miRNAs can mediate that process. The presence or altered levels of miRNA expression could be a possible biomarker for PD.

Serum miRNA Expression

In a study involving 138 patients with PD and 112 control participants, quantitative reverse transcriptase polymerase chain reaction (qRT-PCR) was utilized to measure the expression of 16 serum miRNAs as potential biomarkers [77]. The results indicated that miR-29c, miR-146a, miR-214, and miR-221 were significantly decreased in patients with PD compared to the control group.

Another study identified a panel of five miRNAs that could serve as biomarkers to differentiate PD patients from controls using a qRT-PCR assay [78]. In this study, samples were collected from serum, which is a much less invasive method than obtaining CSF samples through lumbar puncture. The five-miRNA panel was investigated in 61 patients, where miR-195 was significantly elevated in those with PD compared to the control group. In contrast, the other four miRNAs (miR-15b, miR-221, miR-181a, and miR-185) showed decreased expression in PD patients compared to healthy controls.

Plasma miRNA Expression

On the contrary, a study evaluated plasma circulating miRNAs in three independent sets comprising 151 PD patients, 21 MSA patients, and 138 healthy controls using high-throughput RT-PCR [79]. The results showed significant up-regulation of miR-133b and miR-221-3p in early-stage PD, discriminating these patients from controls with 94.4% sensitivity and 91.1% specificity. These miRNAs distinguished PD patients (not limited to early stage) from controls with 84.8% sensitivity and 88.9% specificity, and miR-4454 differentiated PD from MSA with 57.1% sensitivity and 82.6% specificity. A combination of miR-133b, miR-221-3p, and miR-4454 was proposed as a potential non-invasive biomarker for PD diagnosis, distinguishing PD from controls and MSA. These findings contrast with Ma et al. [77], who reported decreased miR-221 in the serum of PD patients, demonstrating challenges in miRNA research, such as variations in biofluids (plasma vs. serum), specific miRNA isoforms (e.g., miR-221-3p vs. miR-221), and methodological differences.

CSF miRNA Expression

CSF miRNAs reflect specific changes in the CNS. A study examined two particular miRNAs, miR-24 and miR-205, in CSF as potential biomarkers for PD [80]. The results showed increased levels of miR-205 and decreased levels of miR-24 in the CSF of PD patients compared to control subjects. These findings suggest that these miRNAs could serve as biomarkers for PD. This highlights the importance of establishing standardized methodologies for analyzing biofluid-specific miRNA profiles.

miRNAs and LRRK2 Mutations

Recent research has concentrated on the relationship between LRRK2 mutations and the expression of miRNAs. A study explored the use of extracellular miRNA signatures to differentiate between LRRK2 mutation carriers and individuals with sporadic PD [81]. The study identified that miR-29c-3p was differentially expressed between these two groups, while miR-425-5p also showed a trend towards significance. Using prediction models based on these miRNA signatures, researchers could accurately predict group affiliation - whether an individual was a LRRK2 mutation carrier or had sporadic PD. Specifically, miRs-128-3p, 29c-3p, 223-3p, and 424-5p were particularly promising discriminators. These findings suggest that LRRK2 mutation status influences extracellular miRNA signatures, and monitoring these signatures could be beneficial in assessing the efficacy of LRRK2-targeting therapies. However, further validation in larger cohorts is necessary to confirm these results.

In summary, varying levels of microRNA expression have been proposed as potential biomarkers for sporadic PD. Table 4 summarizes various findings from studies evaluating miRNA as a potential biomarker in PD. However, the field faces significant challenges related to reproducibility and a lack of consensus, often stemming from methodological differences, the use of different biofluids, and variations in patient cohort characteristics. Utilizing multi-miRNA panels, as demonstrated in several studies, may be a strategic approach to enhance diagnostic accuracy and reliability. While research into the role of miRNAs in PD pathogenesis is ongoing, further studies are needed to understand their clinical utility fully.

**Table 4:** miRNA biomarkers for Parkinson's disease CSF: cerebrospinal fluid; PD: Parkinson's disease; qRT-PCR: quantitative reverse transcription polymerase chain reaction; MSA: multiple system atrophy

Study	Study Subjects	Method Evaluated	Biomarker(s)	Key Findings
Serum miRNA Expression				
Ma et al., 2016 [77]	138 patients with PD and 112 controls.	Quantitative reverse transcriptase polymerase chain reaction (qRT-PCR) to measure expression of 16 serum miRNAs.	Serum miR-29c, miR-146a, miR-214, and miR-221.	MiR-29c, miR-146a, miR-214, and miR-221 were significantly decreased in PD serum compared to controls.
Ding et al., 2016 [78]	61 PD patients and controls (for the 5-miRNA panel validation).	qRT-PCR assay for a panel of five miRNAs in serum.	Serum panel: miR-195, miR-15b, miR-221, miR-181a, and miR-185.	miR-195 was significantly elevated in PD patients versus controls. MiR-15b, miR-221, miR-181a, and miR-185 were decreased in PD patients compared to healthy controls. Panel could discriminate PD patients from controls.
Plasma miRNA Expression				
Chen et al., 2021 [79]	Three independent sets: 151 PD patients, 21 MSA patients, and 138 healthy controls.	High-throughput RT-PCR for plasma circulating miRNAs.	Plasma miR-133b, miR-221-3p, and miR-4454.	Significant up-regulation of miR-133b and miR-221-3p in early-stage PD (94.4% sensitivity, 91.1% specificity vs. controls). These also distinguished PD (all stages) from controls (84.8% sensitivity, 88.9% specificity). miR-4454 differentiated PD from MSA (57.1% sensitivity, 82.6% specificity).
CSF miRNA Expression				
Marques et al., 2017 [80]	PD patients and controls.	Evaluation of miRNA (miR-24 and miR-205) in CSF.	CSF miR-24 and miR-205.	Increased miR-205 and decreased miR-24 in PD patients’ CSF compared to controls.
miRNAs and LRRK2 Mutations				
Braunger et al., 2024 [81]	LRRK2 mutation carriers are individuals with sporadic PD.	Investigation of extracellular miRNA signatures (method implies molecular analysis like qPCR or sequencing).	Extracellular miR-29c-3p, miR-425-5p; Panel: miRs-128-3p, 29c-3p, 223-3p, and 424-5p.	miR-29c-3p differentially expressed between LRRK2 mutation carriers and sporadic PD. miR-425-5p showed a trend. Prediction models based on miRNA signatures (esp. a 4-miRNA panel) predicted group affiliation with high accuracy.

Challenges, standardization, and opportunities for biomarker evaluation in PD

As highlighted, α-Syn, LRRK2, DJ-1, and miRNAs have considerable potential as biomarkers. Nevertheless, their development and clinical validation encounter significant challenges. Validating dependable biomarkers for PD is essential for facilitating early diagnosis, tracking disease progression, and assessing treatment interventions, especially for therapies aimed at modifying the disease. Therefore, implementing thorough and rigorous standardization and utilizing emerging technologies presents transformative opportunities to enhance PD biomarker research.

Challenges in Biomarker Development

Disease heterogeneity and pathophysiological complexity: PD presents a variety of clinical symptoms, including motor symptoms such as tremor, rigidity, and bradykinesia, as well as non-motor symptoms such as constipation, hyposmia, and RBD. This diversity reflects underlying molecular and pathological variability, which includes α-syn aggregation, mitochondrial dysfunction, oxidative stress, and genetic predispositions. This heterogeneity could complicate the identification of a single biomarker applicable across PD subtypes. For example, α-syn levels vary across biofluids and disease stages, with inconsistent blood-based results due to factors such as hemolysis [82]. Similarly, LRRK2 mutations are more prevalent in specific populations, limiting generalizability.

Early and prodromal detection: Significant dopaminergic neuron loss (>50-70% in the substantia nigra) occurs before motor symptoms manifest, necessitating biomarkers sensitive to prodromal stages (e.g., RBD, hyposmia) for timely intervention. While α-syn SAAs detect prodromal PD [16], subtle early changes often overlap with normal aging or other conditions, reducing specificity.

Biofluid and sample variability:* *Variability in biofluids (CSF, blood, saliva, skin, retina) affects biomarker reliability. For instance, hemolysis in blood samples confounds α-syn and DJ-1 measurements due to their high abundance in erythrocytes [24,28]. Saliva samples are susceptible to oral contamination, impacting α-syn detection [38]. Pre-analytical factors, including sample collection, processing, and storage conditions (e.g., centrifugation, freeze-thaw cycles), also contribute to inconsistent results across studies.

Methodological inconsistencies: Variations in assay techniques (e.g., ELISA, Luminex, RT-QuIC, qRT-PCR) and their sensitivity, specificity, and calibration lead to discrepant findings. For DJ-1, contradictory CSF results [28,70] stem from differences in blood contamination controls. Similarly, miRNA studies report conflicting results for miR-221 due to differences in biofluids (serum vs. plasma) and isoforms (miR-221 vs. miR-221-3p) [77,79].

Limited specificity and sensitivity: Many biomarkers lack sufficient specificity to distinguish PD from other α-synucleinopathies (e.g., DLB, MSA) or neurodegenerative disorders. For example, oxidized DJ-1 reflects oxidative stress but is not PD-specific [68]. Total α-syn assays often lack sensitivity, though newer methods targeting specific forms (e.g., phosphorylated α-syn) show promise [42].

Resource-intensive validation: Validating biomarkers requires large-scale, longitudinal studies with diverse, well-characterized cohorts, including prodromal individuals and controls with other Parkinsonian syndromes. These studies are costly and time-consuming, particularly when accounting for genetic variations such as LRRK2 mutations.

Standardization Need

Standardization is essential to enhance biomarker reliability and reproducibility among various ongoing studies and collectively identify the highly specific and sensitive biomarker for PD. This will help overcome the hurdle that limits clinical application.

Standardized operating procedures (SOPs): Uniform protocols for biofluid and tissue collection, processing, and storage are critical. These protocols will help minimize variability in sample handling, which is important for obtaining reliable and comparable data across different research facilities and studies. Initiatives such as the Parkinson’s Progression Markers Initiative (PPMI) provide SOPs that can minimize variability.

Assay harmonization, reference standards, and data sharing: Standardizing assay methodologies, including antibody specificity, detection limits, and calibration, is necessary. For example, α-syn SAAs require consistent protocols to maintain high sensitivity and specificity [21,22]. Additionally, establishing reference ranges for biomarker levels in PD and controls and secure data-sharing platforms can facilitate meta-analyses.

Cohort characterization: Standardizing clinical and diagnostic criteria across cohorts, including age, disease stage, and genetic background, ensures comparability. Including diverse populations and prodromal groups (e.g., RBD, LRRK2 carriers) will enhance the generalizability of the findings.

Opportunities for Advancing Biomarker Evaluation

Emerging technologies and strategies offer significant opportunities to overcome the above challenges.

Advanced assay technologies: Ultrasensitive techniques, such as α-syn [23], RT-QuIC for skin and saliva [39,43], and SISCAPA for LRRK2 [54], can enhance the detection of low-abundance pathological forms, thereby increasing sensitivity for diagnosing prodromal and early stages of PD.

Multi-biomarker panels: Combining the biomarkers for which there is evidence of the PD association could enhance diagnostic accuracy. Several studies have reported robustness, improving early diagnosis and differentiation among α-synucleinopathies and disease correlation. For example, pairing salivary α-syn with miRNA-29a-3p has been shown to improve PD vs. MSA differentiation [39], and multi-miRNA panels demonstrated enhanced robustness [78].

Non-invasive matrices: Non-invasive sample sources such as saliva, skin, and retinal scans can simplify collection, reduce patient burden, and improve compliance. These methods may increase participation in large cohort studies, enabling better insights into PD. If these sources yield sensitive and specific biomarkers for early and differential diagnosis of PD and α-synucleinopathies, these methods could serve as practical tools for clinical use.

Advanced omics technologies: High-throughput genomics, transcriptomics (e.g., miRNA profiling), proteomics, and metabolomics could facilitate the identification of novel biomarker signatures. For instance, miRNA panels [79] and proteomic analyses of CSF could identify complementary markers.

Prodromal and pharmacodynamic biomarkers: As highlighted, biomarkers such as α-syn SAA [23] and LRRK2-related DaT deficits [58] could facilitate prodromal detection, while longitudinal markers such as pT73-Rab10 in urinary EVs [57] could aid in therapeutic monitoring.

Numerous opportunities exist to identify and overcome avoidable challenges through optimizing and standardizing studies. This approach can enhance the evaluation of studies and identify potential biomarkers from large cohorts across various multicenter studies.

Polygenic Risk Scores (PRS)

While established biomarkers such as α-synuclein, LRRK2, DJ-1, and miRNAs remain central to PD research, recent advancements are expanding the landscape of diagnostic and prognostic tools. These emerging areas offer novel insights into disease mechanisms, risk identification, and progression tracking.

PRS are valuable prognostic tools that aggregate genome-wide significant variants, quantifying cumulative genetic risk and aiding in the identification of individuals at high risk for complex diseases, including PD [83]. Recent studies using various genome-wide association studies (GWAS) have established general PRS models that can discriminate PD patients from neurologically non-carriers [84-86]. For example, in clinically defined PD, PRS significantly modified disease risk in both GBA1 mutation carriers and non-carriers and demonstrated a trend in LRRK2 G2019S carriers. The association between PRS and PD risk in LRRK2 carriers was primarily driven by the rs7938782-A risk allele [87]. Additionally, PRS was negatively correlated with age-at-onset in non-carriers, suggesting its potential role in disease progression.

However, a standalone PRS for PD has limited clinical utility, with reported sensitivity of 56.9% and specificity of 63.2%, which restricts its effectiveness as a sole diagnostic tool [88]. Importantly, the utility of PRS improves when integrated with clinical data such as olfactory function, family history, age, and gender [88]. Moreover, combining PRS with above mentioned biomarkers or neuroimaging may further enhance its diagnostic and prognostic value.

Ongoing clinical trials evaluating biomarkers in PD

Recent advancements have spurred various clinical trials exploring diverse biomarker modalities, including retinal measurements, serum metabolites, phosphorylated alpha-synuclein in skin biopsies, and slow wave sleep (SWS). These studies aim to enhance diagnostic accuracy, track disease progression, and inform personalized therapeutic strategies. To illustrate the scope of current research, Table 5 summarizes selective active clinical trials evaluating various aspects of biomarker development for PD, sourced from ClinicalTrials.gov as of June 2025.

**Table 5:** Ongoing clinical trials that are evaluating biomarkers in Parkinson’s disease DLB: dementia with Lewy bodies; MSA: multiple system atrophy; PD: Parkinson’s disease; PMCA: protein misfolding cyclic amplification; RT-QuIC: real-time quaking-induced conversion

Study NCT Number	Study Type	Participants	Purpose/Objective of the Study	Primary End Points
NCT06929026 [89]	Observational (Patient Registry)	~60 adults (18+ years) with PD stages 0–3 (Hoehn and Yahr scale) and age-/gender-matched healthy controls.	To determine if retinal vessel oxyhemoglobin saturation, vessel width, retinal nerve fiber layer thickness, and ganglion cell/visual cortex responses can serve as biomarkers for PD.	Retinal vessel oxyhemoglobin saturation (measured by retinal oximetry at baseline).
NCT05769699 [90]	Interventional (Single Group Assignment)	~200 adults (18–80 years) with idiopathic PD per Movement Disorder Society (MDS) criteria.	To characterize clinical features and measure serum biomarkers (neurodegeneration, synaptic, and inflammation) in PD patients at various stages to understand disease progression.	Clinical characteristics via MDS-UPDRS and NMS score changes, and serum levels of IL-1β, IL-6, IL-5, IL-4, IL-17, TNF-α, IFN-γ, IL-10, α-Syn, NfL, BDNF (measured over 3 years).
NCT05385315 [91]	Observational (Cohort, Prospective)	~70 adults (18+ years) with de novo PD, advanced PD (>5 years, treated), or Multiple System Atrophy (MSA).	To validate a metabolic biomarker (six serum metabolites: acetoacetate, betaine, beta-hydroxybutyrate, creatine, pyruvate, valine) for early PD diagnosis and differentiate PD from MSA.	Accuracy of the biomarker in classifying advanced PD patients (>5 years, L-Dopa treated) against clinical diagnosis (measured over 2 years).
NCT04796506 [92]	Interventional (Randomized, Sequential Assignment)	~120 adults (45+ years) with idiopathic PD (Hoehn and Yahr stages 2–3, MoCA 18–25).	To investigate the effects of progressive resistance training (PRT) on cognition, evaluate slow wave sleep (SWS) as a biomarker/mediator of cognitive improvement, and optimize rehabilitation by switching non-responders to endurance training (ET).	Change in executive function measured by the Stroop Inhibition test (baseline to 12 weeks).
NCT06621602 [93]	Observational (Cohort, Prospective)	~100 adults (50–100 years) with PD (75) or REM Sleep Behavior Disorder (RBD) (25).	To assess p-α-syn levels in skin biopsies as a biomarker for PD and RBD progression, tracking p-α-syn deposition over time.	Accuracy, precision, and sensitivity of the pathological test in detecting p-α-Syn aggregation/deposition in skin biopsies, and changes in p-α-Syn levels correlated with clinical assessments (measured over 18 months).
NCT06232772 [94]	Observational (Cohort, Cross-Sectional)	~600 adults (18–80 years) with clinically defined PD, clinically probable PD, MSA, PSP, and age-/sex-matched healthy subjects.	To evaluate the detection ability of the α-syn ultrafine fluorescence detection method in body fluids (saliva, urine, cerebrospinal fluid, blood) and skin for early diagnosis and differential diagnosis of PD, MSA, and other α-synucleinopathies.	Accuracy, precision, sensitivity, and specificity of skin biopsy and body fluid detection of α-Syn Ultrafine Fluorescence Method (measured over 3 years).
NCT04518059 [95]	Observational (Cohort, Prospective)	~250 adults (21–89 years) with PD, MSA, DLB, PSP, CBD, or healthy controls.	To determine if misfolded α-syn in skin punch biopsies can differentiate α-Synucleinopathies (PD, MSA, DLB) from tauopathies (PSP, CBD) and healthy controls for early diagnosis of Parkinsonism.	Amount of alpha-synuclein in skin measured by RT-QuIC and sPMCA (cross-sectional at baseline).

## Conclusions

The exploration of biomarkers for PD reveals significant promise in α-syn, LRRK2, DJ-1, and miRNAs, each offering unique insights into PD pathogenesis and potential for early diagnosis and disease monitoring. α-Syn, central to PD’s hallmark Lewy bodies, shows high sensitivity and specificity in advanced assays such as SAAs in CSF, blood, and saliva, particularly for detecting pathological forms and prodromal stages. LRRK2, linked to familial and sporadic PD, provides genetic, CSF, and peripheral blood markers, with assays such as pT73-Rab10 phosphorylation enhancing its utility for therapy monitoring. DJ-1, particularly in its oxidized and isoform-specific forms, reflects oxidative stress in PD across urine, CSF, and plasma exosomes, though inconsistencies necessitate standardized assays. miRNAs, including miR-29c, miR-133b, and miR-221-3p, offer non-invasive diagnostic potential in serum, plasma, and CSF, with multi-miRNA panels improving accuracy despite biofluid and methodological variability challenges. Collectively, these biomarkers facilitate earlier detection, patient stratification, and therapeutic evaluation, particularly for disease-modifying therapies. However, their clinical application is hindered by reproducibility issues, biofluid-specific differences, and the need for large-scale validation. Ongoing research and standardization efforts are critical to establishing sensitive, specific, and non-invasive biomarkers to improve PD diagnosis, differentiation from other α-synucleinopathies, and patient outcomes.
